# Supplementary material for: Effect modification of diabetic status on the association between exposure to particulate matter and cardiac arrhythmias in a general population: A systematic review and meta-analysis
Source: PLoS One. 2024 May 17;19(5):e0301766. doi: 10.1371/journal.pone.0301766 (PMC11101100; doi:10.1371/journal.pone.0301766)
Supplement: S1 File — (PDF) [file pone.0301766.s002.pdf]

National Institute for Health Research

UNIVERSITY of York
Centre for Reviews and Dissemination

Systematic review

A list of fields that can be edited in an update can be found here

1. * Review title.

Give the title of the review in English

Effect modification of diabetic status on the association between exposure to particulate matter and cardiac arrhythmias: a systematic review and meta-analysis

2. Original language title.

For reviews in languages other than English, give the title in the original language. This will be displayed with the English language title.

3. * Anticipated or actual start date.

Give the date the systematic review started or is expected to start.

18/01/2023

4. * Anticipated completion date.

Give the date by which the review is expected to be completed.

18/07/2023

5. * Stage of review at time of this submission.

This field uses answers to initial screening questions. It cannot be edited until after registration.

Tick the boxes to show which review tasks have been started and which have been completed.

Update this field each time any amendments are made to a published record.

The review has not yet started: No

Review stage	Started	Completed
Preliminary searches	Yes	No
Piloting of the study selection process	Yes	No
Formal screening of search results against eligibility criteria	Yes	No
Data extraction	No	No
Risk of bias (quality) assessment	No	No
Data analysis	No	No

Provide any other relevant information about the stage of the review here.

6. * Named contact.

The named contact is the guarantor for the accuracy of the information in the register record. This may be any member of the review team.

Kiattichat Tassanaviroj

Email salutation (e.g. "Dr Smith" or "Joanne") for correspondence:

Mr Tassanaviroj

7. * Named contact email.

Give the electronic email address of the named contact.

kiattichat_t@cmu.ac.th

8. Named contact address

Give the full institutional/organisational postal address for the named contact.

110 Faculty of Medicine, CMU, Inthawarorot Rd., Sri Phum, Muang, Chiang Mai 50200

9. Named contact phone number.

Give the telephone number for the named contact, including international dialling code.

+66611324562

10. * Organisational affiliation of the review.

Full title of the organisational affiliations for this review and website address if available. This field may be

completed as 'None' if the review is not affiliated to any organisation.

Chiang Mai University

Organisation web address:

https://www.med.cmu.ac.th

11. * Review team members and their organisational affiliations.

Give the personal details and the organisational affiliations of each member of the review team. Affiliation refers to groups or organisations to which review team members belong. **NOTE: email and country now MUST be entered for each person, unless you are amending a published record.**

Mr Kiattichat Tassanaviroj. Faculty of Medicine, Chiang Mai University.
Miss Pimchanok Plodphai. Faculty of Medicine, Chiang Mai University.
Dr Pakpoom Wongyikul. Faculty of Medicine, Chiang Mai University.
Dr Krittai Tanasombatkul. Faculty of Medicine, Chiang Mai University.
Assistant/Associate Professor Krekwit Shinlapawittayatorn. Faculty of Medicine, Chiang Mai University.
Dr Phichayut Phinyo. Faculty of Medicine, Chiang Mai University.

12. * Funding sources/sponsors.

Details of the individuals, organizations, groups, companies or other legal entities who have funded or sponsored the review.

None

Grant number(s)

State the funder, grant or award number and the date of award

None

13. * Conflicts of interest.

List actual or perceived conflicts of interest (financial or academic).

None

14. Collaborators.

Give the name and affiliation of any individuals or organisations who are working on the review but who are not listed as review team members. **NOTE: email and country must be completed for each person, unless you are amending a published record.**

15. * Review question.

State the review question(s) clearly and precisely. It may be appropriate to break very broad questions down into a series of related more specific questions. Questions may be framed or refined using PI(E)COS or similar where relevant.

1. To evaluate the effect of particulate matter on arrhythmias.

2. To compare the occurrence of cardiac arrhythmia in the presence of particulate matter between diabetic and non-diabetic patients.

16. * Searches.

State the sources that will be searched (e.g. Medline). Give the search dates, and any restrictions (e.g. language or publication date). Do NOT enter the full search strategy (it may be provided as a link or attachment below.)

Limited to: cohort and case-crossover study.

Databases: PubMed and Embase, from the inception to the search date.

Language restriction: published in English only.

Re-run: prior to the final analysis.

The key search strategy: (particulate matter) AND (arrhythmia) AND (diabetes).

17. URL to search strategy.

Upload a file with your search strategy, or an example of a search strategy for a specific database, (including the keywords) in pdf or word format. In doing so you are consenting to the file being made publicly accessible. Or provide a URL or link to the strategy. Do NOT provide links to your search **results**.

Alternatively, upload your search strategy to CRD in pdf format. Please note that by doing so you are consenting to the file being made publicly accessible.

Do not make this file publicly available until the review is complete

18. * Condition or domain being studied.

Give a short description of the disease, condition or healthcare domain being studied in your systematic review.

General population

19. * Participants/population.

Specify the participants or populations being studied in the review. The preferred format includes details of both inclusion and exclusion criteria.

Patients with and without diabetes mellitus

20. * Intervention(s), exposure(s).

Give full and clear descriptions or definitions of the interventions or the exposures to be reviewed. The preferred format includes details of both inclusion and exclusion criteria.

Higher exposure of Particulate matter (PM2.5 and PM10)

21. * Comparator(s)/control.

Where relevant, give details of the alternatives against which the intervention/exposure will be compared (e.g. another intervention or a non-exposed control group). The preferred format includes details of both inclusion and exclusion criteria.

Lower exposure to particulate matter (PM2.5 and PM10)

22. * Types of study to be included.

Give details of the study designs (e.g. RCT) that are eligible for inclusion in the review. The preferred format includes both inclusion and exclusion criteria. If there are no restrictions on the types of study, this should be stated.

cohort, case-crossover study

23. Context.

Give summary details of the setting or other relevant characteristics, which help define the inclusion or exclusion criteria.

Studies took place in any country, report outcome of hospital admissions, emergency room visits, and ECG measurements.

24. * Main outcome(s).

Give the pre-specified main (most important) outcomes of the review, including details of how the outcome is defined and measured and when these measurement are made, if these are part of the review inclusion criteria.

Any cardiac arrhythmias (hospital admissions, ER visits, ECG measurement)

Measures of effect

Please specify the effect measure(s) for you main outcome(s) e.g. relative risks, odds ratios, risk difference, and/or 'number needed to treat.

Odds ratios

25. * Additional outcome(s).

List the pre-specified additional outcomes of the review, with a similar level of detail to that required for main outcomes. Where there are no additional outcomes please state 'None' or 'Not applicable' as appropriate to the review

None

Measures of effect

Please specify the effect measure(s) for you additional outcome(s) e.g. relative risks, odds ratios, risk difference, and/or 'number needed to treat.

None

26. * Data extraction (selection and coding).

Describe how studies will be selected for inclusion. State what data will be extracted or obtained. State how this will be done and recorded.

We will search the titles and abstracts of relevant literature from the databases until the end of December 2022. Two screening procedures will be employed in this review. For the first procedure, two investigators (K.T. and P.P.) will then screen records for relevant evidence using Rayyan, a web application to screen systematic review records. For the second procedure, an open-source machine learning entitled ASReview will be used for priority screening. ASReview will be given three relevant and three irrelevant inputs to learn to rearrange the records according to their relevance to the inputs. One investigator (K.T.) will screen through the rearranged records using ASReview. The records screening will be stopped after the investigators have screened 50% of the records (a pre-specified stopping rule). The results of the two screening procedures will be combined and discussed. Any disagreement during the screening process will be resolved by consulting with a clinical expert in cardiac electrophysiology (K.S.).

The following information will be independently extracted from each study by two trained investigators (K.T. and P.P.): study authorship, year of publication, study period, country/location, study design, patient demographics including mean age, sample size, types of particulate matter, types of effect (i.e., short, or long term), outcomes (i.e., hospital admission, or ER visit), and Odds ratio. Any discrepancy during the data extraction was resolved through discussion with the cardiac electrophysiology expert (K.S.).

For studies with incomplete outcome data, we will contact the corresponding authors of those studies via email. If the corresponding authors did not respond within 2 weeks, the data would be imputed (if feasible) or reported as missing. For dichotomous outcomes, we will extract the odds ratios and their 95% confidence interval.

27. * Risk of bias (quality) assessment.

State which characteristics of the studies will be assessed and/or any formal risk of bias/quality assessment tools that will be used.

Two authors (K.T. and P.P.) independently evaluated the quality of each included study using the Newcastle-Ottawa Scale (NOS) quality assessment tool. NOS assessed the quality of study and potential bias based on three separate domains, which are subject selection, comparability, and outcome assessment. According to the number of stars received within each domain, studies will be rated as good, fair, or poor quality. Studies with 3 or 4 stars in the selection domain, 1 or 2 stars in the comparability domain, and 2 or 3 stars in the outcome domain will be rated as good quality. Studies with 2 stars in the selection domain, 1 or 2 stars in the comparability domain, and 2 or 3 stars in the outcome domain will be rated as fair quality. Studies with 0 or 1 stars in the selection domain, or 0 stars in the comparability domain, or 0 or 1 stars in the outcome domain will be rated as poor quality. Any disagreement will be resolved during the quality assessment process by consulting with the clinical expert in cardiac electrophysiology (K.S.) and the clinical methodologist (P.P.).

28. * Strategy for data synthesis.

Describe the methods you plan to use to synthesise data. This **must not be generic text** but should be **specific to your review** and describe how the proposed approach will be applied to your data. If meta-analysis is planned, describe the models to be used, methods to explore statistical heterogeneity, and software package to be used.

A traditional approach of a pairwise meta-analysis will be used for data synthesis. The statistical heterogeneity will be evaluated using the Cochrane Q test and I² statistics. As all studies are expected to carry clinical and methodological heterogeneity, we use the DerSimonian and Laird random-effects model to estimate the pooled estimates with their corresponding 95% confidence intervals (CIs). Due to the variation in the scoring components, the primary endpoint of interest, we plan to pool the estimates from all studies using Odds ratio (OR)

This systematic review and meta-analysis will be conducted and reported in concordance with the Cochrane Handbook of Systematic Review and the Preferred Reporting Items for Systematic Reviews and Meta-analyses (PRISMA), respectively. All analyses will be performed using Stata 17 (StataCorp, College Station, Texas, USA). P-values 0.05 will be considered statistically significant.

29. * Analysis of subgroups or subsets.

State any planned investigation of ‘subgroups’. Be clear and specific about which type of study or participant will be included in each group or covariate investigated. State the planned analytic approach.

We will perform a subgroup analysis of diabetic status to investigate whether diabetic status modify the effect of the association between exposure to particulate matter and cardiac arrhythmias.

30. * Type and method of review.

Select the type of review, review method and health area from the lists below.

Type of review

Cost effectiveness

No

Diagnostic

No

Epidemiologic

No

Individual patient data (IPD) meta-analysis

No

Intervention

No

Living systematic review

No

Meta-analysis

Yes

Methodology

No

Narrative synthesis

No

Network meta-analysis

No

Pre-clinical

No

Prevention

No

Prognostic

No

Prospective meta-analysis (PMA)

No

Review of reviews

No

Service delivery

No

Synthesis of qualitative studies

No

Systematic review

Yes

Other

No

Health area of the review

Alcohol/substance misuse/abuse

No

Blood and immune system

No

Cancer

No

Cardiovascular

Yes

Care of the elderly

No

Child health

No

Complementary therapies

No

COVID-19

No

Crime and justice

No

Dental

No

Digestive system

No

Ear, nose and throat

No

Education

No

Endocrine and metabolic disorders

No

Eye disorders

No

General interest

No

Genetics

No

Health inequalities/health equity

No

Infections and infestations

No

International development

No

Mental health and behavioural conditions

No

Musculoskeletal

No

Neurological

No

Nursing
No

Obstetrics and gynaecology
No

Oral health
No

Palliative care
No

Perioperative care
No

Physiotherapy
No

Pregnancy and childbirth
No

Public health (including social determinants of health)
No

Rehabilitation
No

Respiratory disorders
No

Service delivery
No

Skin disorders
No

Social care
No

Surgery
No

Tropical Medicine
No

Urological
No

Wounds, injuries and accidents
No

Violence and abuse

No

31. Language.

Select each language individually to add it to the list below, use the bin icon to remove any added in error.

English

There is not an English language summary

32. * Country.

Select the country in which the review is being carried out. For multi-national collaborations select all the countries involved.

Thailand

33. Other registration details.

Name any other organisation where the systematic review title or protocol is registered (e.g. Campbell, or The Joanna Briggs Institute) together with any unique identification number assigned by them. If extracted data will be stored and made available through a repository such as the Systematic Review Data Repository (SRDR), details and a link should be included here. If none, leave blank.

34. Reference and/or URL for published protocol.

If the protocol for this review is published provide details (authors, title and journal details, preferably in Vancouver format)

Add web link to the published protocol.

Or, upload your published protocol here in pdf format. Note that the upload will be publicly accessible.

No I do not make this file publicly available until the review is complete

Please note that the information required in the PROSPERO registration form must be completed in full even if access to a protocol is given.

35. Dissemination plans.

Do you intend to publish the review on completion?

No

Give brief details of plans for communicating review findings.?

The full report will be submitted to a journal in the field of cardiology, or environmental health.

36. Keywords.

Give words or phrases that best describe the review. Separate keywords with a semicolon or new line. Keywords help PROSPERO users find your review (keywords do not appear in the public record but are included in searches). Be as specific and precise as possible. Avoid acronyms and abbreviations unless these are in wide use.

particulate matter; cardiac arrhythmia; diabetes mellitus

37. Details of any existing review of the same topic by the same authors.

If you are registering an update of an existing review give details of the earlier versions and include a full bibliographic reference, if available.

Not applicable

38. * Current review status.

Update review status when the review is completed and when it is published.New registrations must be ongoing so this field is not editable for initial submission.

Please provide anticipated publication date

Review_Ongoing

39. Any additional information.

Provide any other information relevant to the registration of this review.

We focus specifically on the effect modification of diabetes

40. Details of final report/publication(s) or preprints if available.

Leave empty until publication details are available OR you have a link to a preprint (NOTE: this field is not editable for initial submission). List authors, title and journal details preferably in Vancouver format.

Give the link to the published review or preprint.
