# Supplementary material for: Effect modification of diabetic status on the association between exposure to particulate matter and cardiac arrhythmias in a general population: A systematic review and meta-analysis
Source: PLoS One. 2024 May 17;19(5):e0301766. doi: 10.1371/journal.pone.0301766 (PMC11101100; doi:10.1371/journal.pone.0301766)
Supplement: S2 File — (DOCX) [file pone.0301766.s003.docx]

**Supporting information**

**
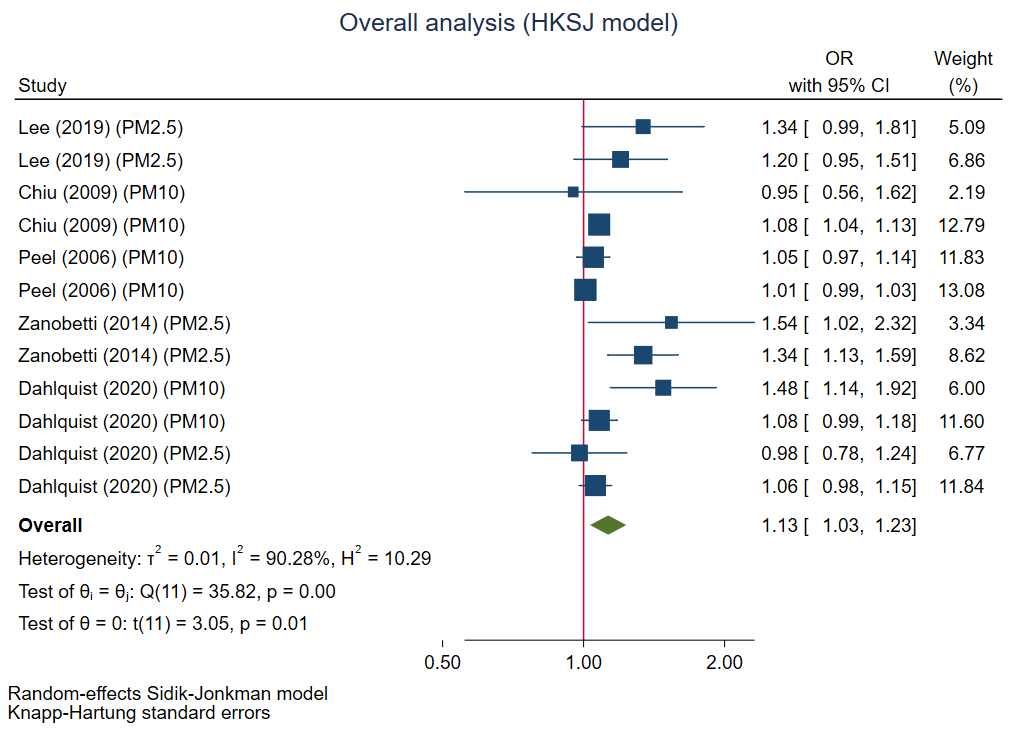
**

**S1 Fig The pooled association between PM exposure and cardiac arrhythmias in the overall population using Hartung-Knapp-Sidak-Jonkman method.** The effect parameters are expressed as odds ratio (OR) of higher PM exposure over lower PM exposure.


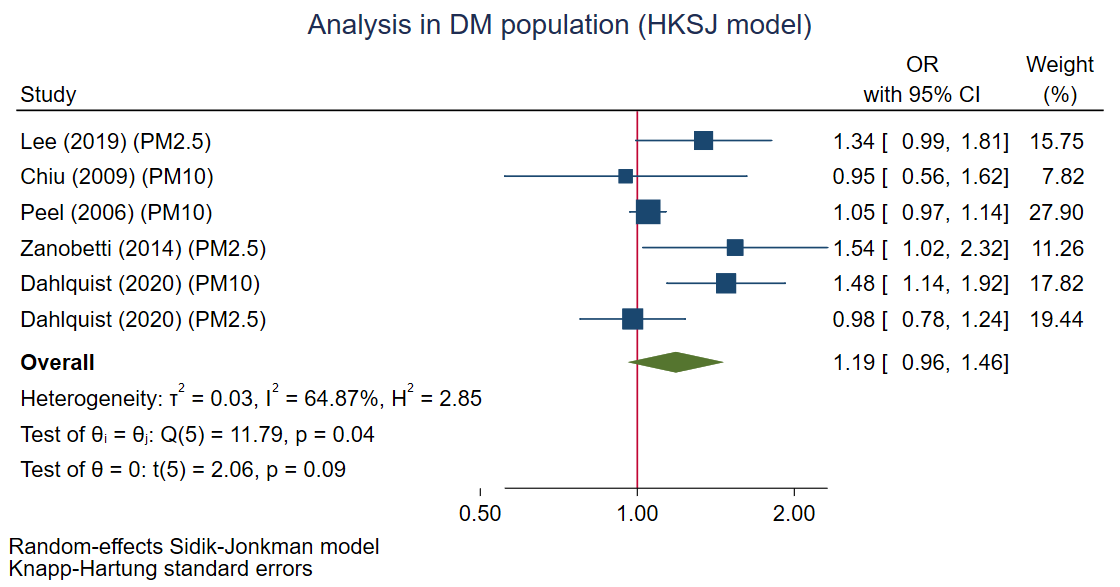


**S2 Fig The pooled association between PM exposure and cardiac arrhythmias in non-diabetic population using Hartung-Knapp-Sidak-Jonkman method.** The effect parameters are expressed as odds ratio (OR) of higher PM exposure over lower PM exposure.

**
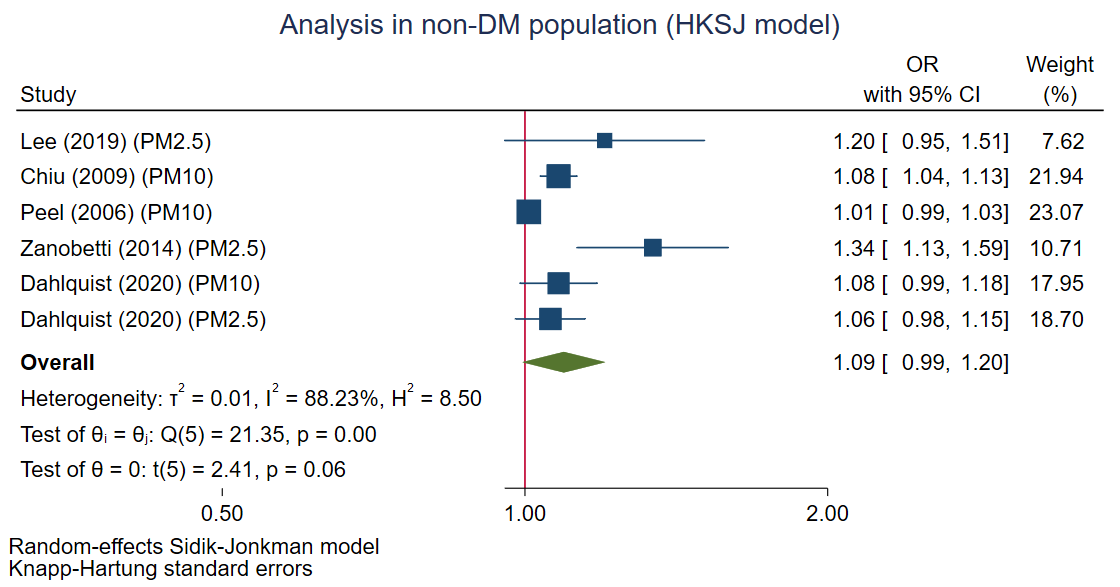
**

**S3 Fig The pooled association between PM exposure and cardiac arrhythmias in diabetic population using Hartung-Knapp-Sidak-Jonkman method.** The effect parameters are expressed as odds ratio (OR) of higher PM exposure over lower PM exposure.

**
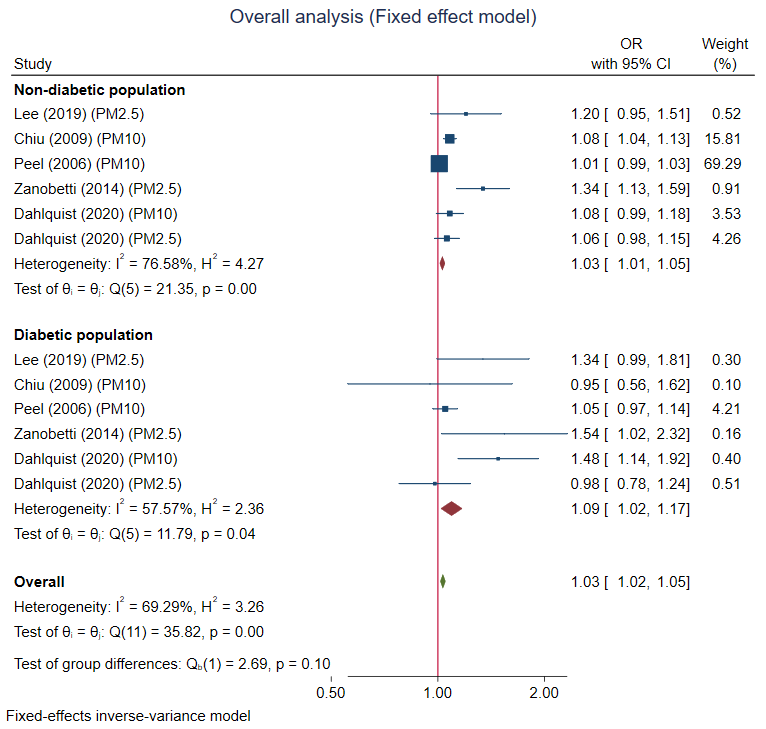
**

**S4 Fig The pooled association between PM exposure and cardiac arrhythmias in the overall population with a subgroup analysis using Fixed-effect model.** The effect parameters are expressed as odds ratio (OR) of higher PM exposure over lower PM exposure.

**
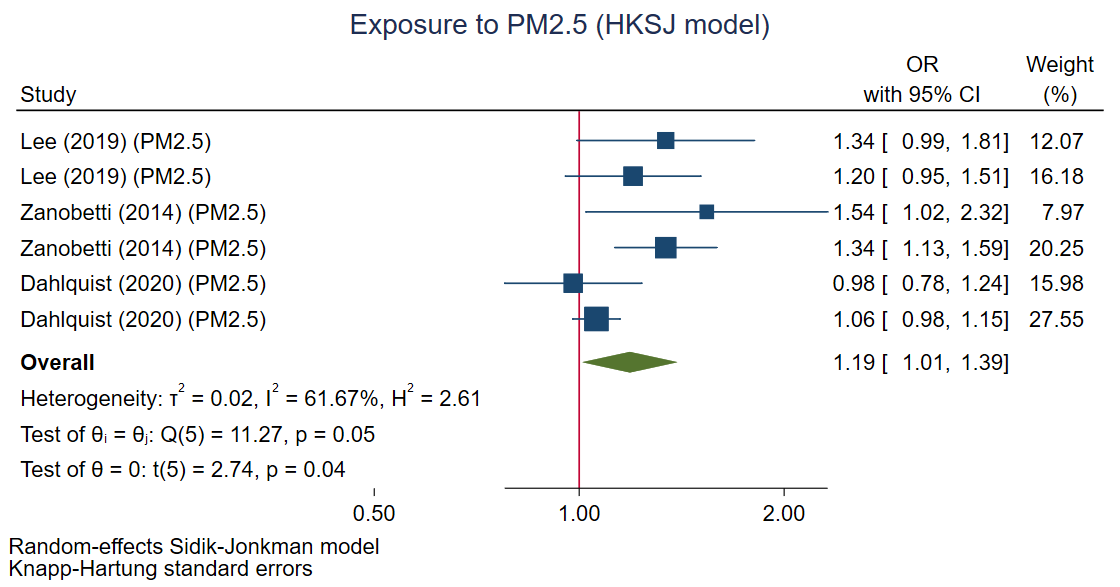
S5 Fig The pooled association between PM2.5 exposure and cardiac arrhythmias in the overall population with a subgroup analysis using Hartung-Knapp-Sidak-Jonkman method.** The effect parameters are expressed as odds ratio (OR) of higher PM2.5 exposure over lower PM2.5 exposure.

**
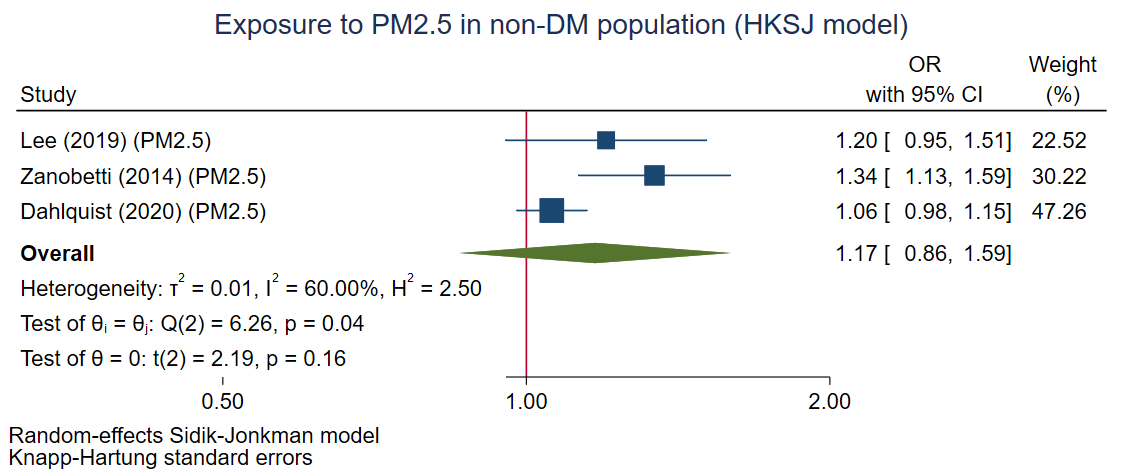
**

**S6 Fig The pooled association between PM2.5 exposure and cardiac arrhythmias in non-diabetic population using Hartung-Knapp-Sidak-Jonkman method.** The effect parameters are expressed as odds ratio (OR) of higher PM2.5 exposure over lower PM2.5 exposure.


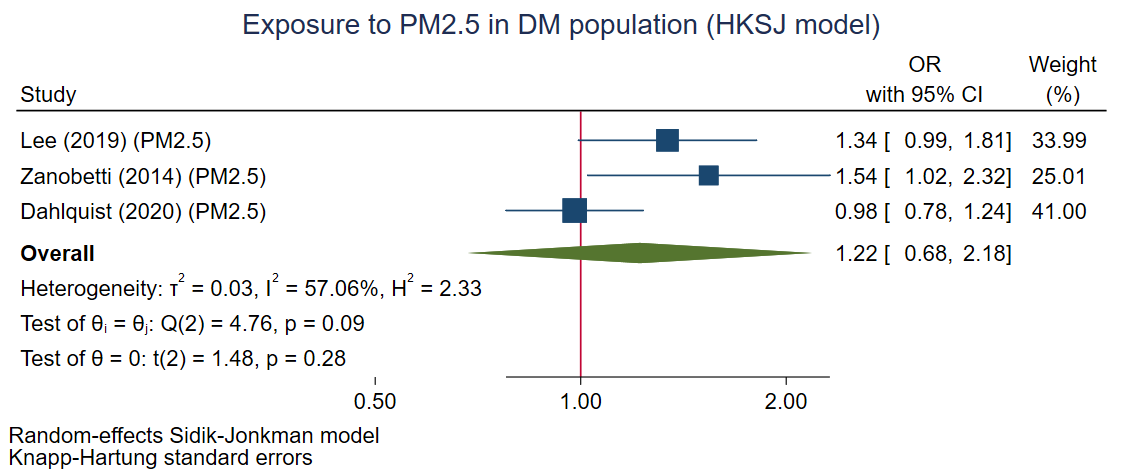


**S7 Fig The pooled association between PM2.5 exposure and cardiac arrhythmias in diabetic population using Hartung-Knapp-Sidak-Jonkman method.** The effect parameters are expressed as odds ratio (OR) of higher PM2.5 exposure over lower PM2.5 exposure.

**
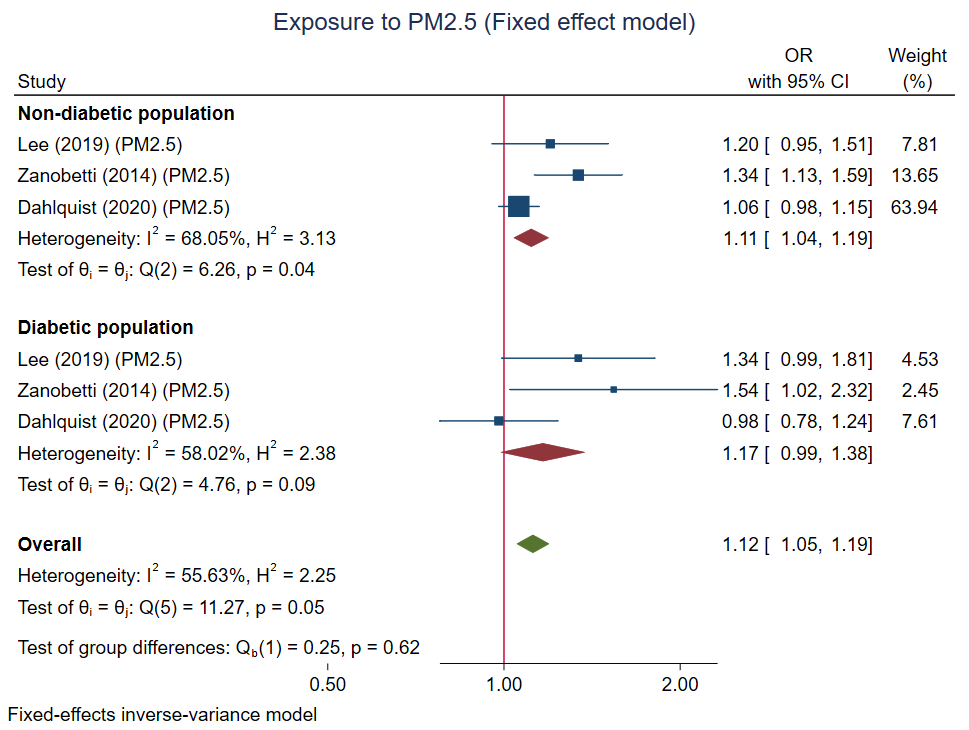
**

**S8 Fig The pooled association between PM2.5 exposure and cardiac arrhythmias in the overall population with a subgroup analysis using Fixed-effect model.** The effect parameters are expressed as odds ratio (OR) of higher PM2.5 exposure over lower PM2.5 exposure.


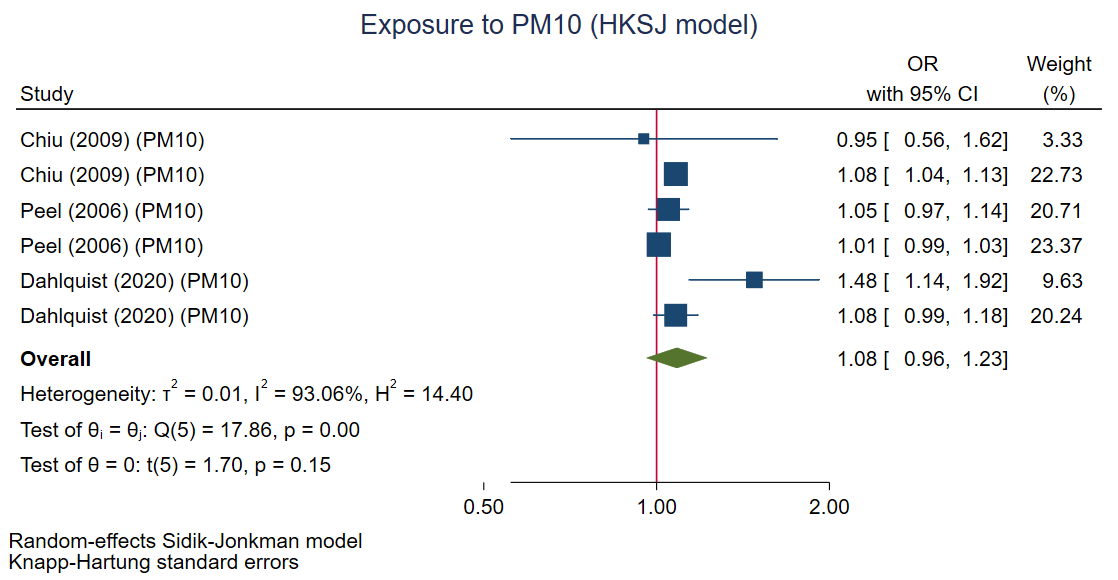


**S9 Fig The pooled association between PM10exposure and cardiac arrhythmias in the overall population using Hartung-Knapp-Sidak-Jonkman method.** The effect parameters are expressed as odds ratio (OR) of higher PM10 exposure over lower PM10 exposure.

**
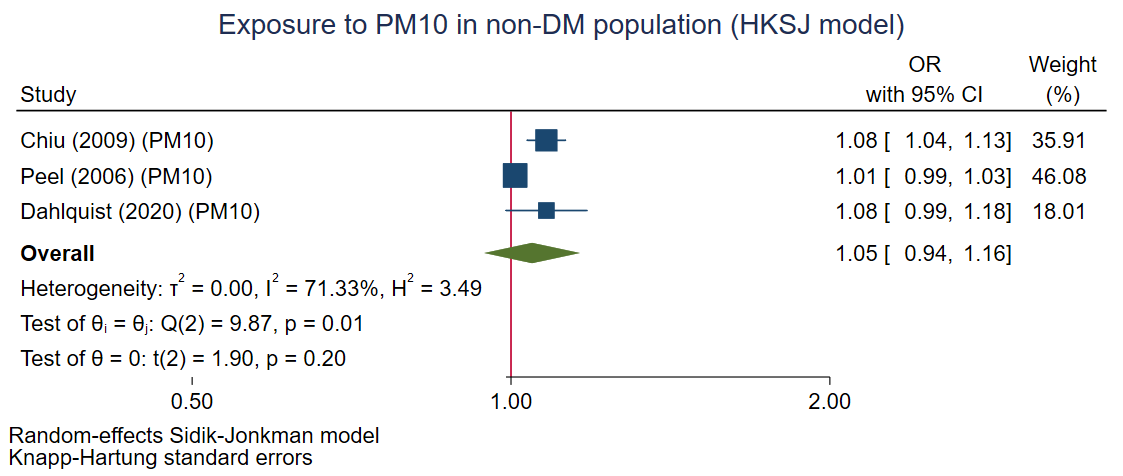
**

**S10 Fig The pooled association between PM10 exposure and cardiac arrhythmias in non-diabetic population using Hartung-Knapp-Sidak-Jonkman method.** The effect parameters are expressed as odds ratio (OR) of higher PM10 exposure over lower PM10 exposure.

**
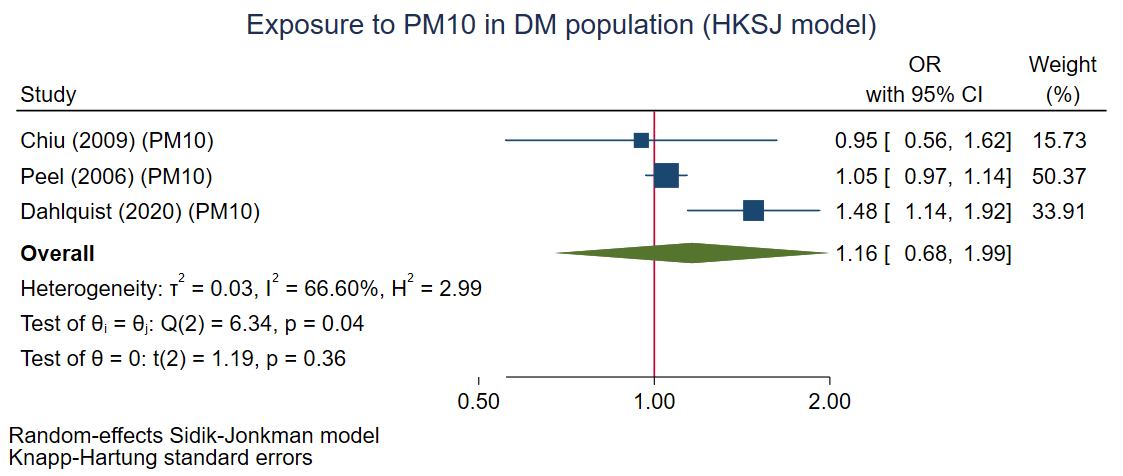
**

**S11 Fig The pooled association between PM10 exposure and cardiac arrhythmias in diabetic population using Hartung-Knapp-Sidak-Jonkman method.** The effect parameters are expressed as odds ratio (OR) of higher PM10 exposure over lower PM10 exposure.

**
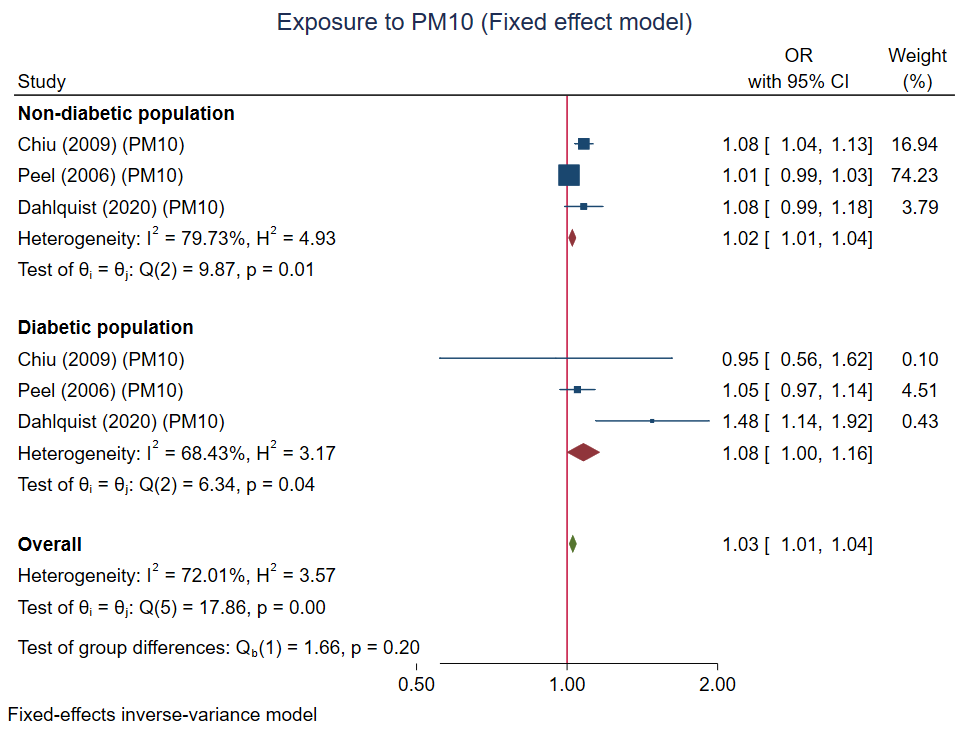
**

**S12 Fig The pooled association between PM10 exposure and cardiac arrhythmias in the overall population with a subgroup analysis using Fixed-effect model.** The effect parameters are expressed as odds ratio (OR) of higher PM10 exposure over lower PM10 exposure.
