# Supplementary material for: Effect modification of diabetic status on the association between exposure to particulate matter and cardiac arrhythmias in a general population: A systematic review and meta-analysis
Source: PLoS One. 2024 May 17;19(5):e0301766. doi: 10.1371/journal.pone.0301766 (PMC11101100; doi:10.1371/journal.pone.0301766)
Supplement: Attachment — Submitted filename: PONE-D-23-26753- With comments.pdf [file pone.0301766.s005.pdf]

PLOS ONE

Effect modification of diabetic status on the association between exposure to particulate matter and cardiac arrhythmias: a systematic review and meta-analysis

--Manuscript Draft--

Manuscript Number:	PONE-D-23-26753
Article Type:	Research Article
Full Title:	Effect modification of diabetic status on the association between exposure to particulate matter and cardiac arrhythmias: a systematic review and meta-analysis
Short Title:	Effect Modification of DM on PM and Arrhythmias
Corresponding Author:	Phichayut Phinyo<br>Chiang Mai University<br>Chiang Mai, Chiang Mai THAILAND
Keywords:	particulate matter; Diabetes; Arrhythmias; Association
Abstract:	Particulate matter (PM) leads to numerous health effects including cardiac arrhythmias. An increasing proportion of population around the world were diagnosed with diabetes mellitus (DM). Exposure to PM and DM have been associated with increased the risk of cardiovascular diseases. Thus, the objective of this study is to investigate whether PM associates with cardiac arrhythmias and whether DM status modify its effect. The search was done on Pubmed and Embase until 18 January 2023. We included cohort and case-crossover studies which showed the effect of PM on cardiac arrhythmias and effect modification of DM status. We used the DerSimonian and Laird random-effects model to calculate the pooled estimates with their corresponding 95% confidence intervals (CIs). We found 217 studies which were subsequently screened. Nine studies matched the inclusion criteria and 5 of them were included in meta-analysis. There were 4,431,452 participants, 2,556 of them had DM. We found that PM, both PM2.5 and PM10, showed significant effect on cardiac arrhythmias (OR 1.10 (95%CI 1.04-1.16)). However, the effect modification of diabetic status was not identified (OR 1.18 (95%CI 1.01-1.38) for diabetic group and OR 1.08 (95%CI 1.02-1.14) for non-diabetic group) (p-value of subgroup difference=0.304). In conclusion, PM is associated with cardiac arrhythmias, but DM status may not modify its effect. The important limitation was the comparability of studies. Further study with good design and methodology is required.
Order of Authors:	Kiattichat Tassanaviroj
	Pimchanok Plodphai
	Pakpoom Wongyikul
	Krittai Tanasombatkul
	Krekwit Shinlapawittayatorn
	Phichayut Phinyo
Additional Information:	
Question	Response
Financial Disclosure<br><br>Enter a financial disclosure statement that describes the sources of funding for the work included in this submission. Review the submission guidelines for detailed requirements. View published research articles from PLOS ONE for specific examples.	The author(s) received no specific funding for this work.

Powered by Editorial Manager® and ProduXion Manager® from Aries Systems Corporation

This statement is required for submission and will appear in the published article if the submission is accepted. Please make sure it is accurate.<br><br>Unfunded studies<br>Enter: The author(s) received no specific funding for this work.<br><br>Funded studies<br>Enter a statement with the following details:<br>• Initials of the authors who received each award<br>• Grant numbers awarded to each author<br>• The full name of each funder<br>• URL of each funder website<br>• Did the sponsors or funders play any role in the study design, data collection and analysis, decision to publish, or preparation of the manuscript?<br>• NO - Include this sentence at the end of your statement: The funders had no role in study design, data collection and analysis, decision to publish, or preparation of the manuscript.<br>• YES - Specify the role(s) played.<br><br>* typeset	
Competing Interests<br><br>Use the instructions below to enter a competing interest statement for this submission. On behalf of all authors, disclose any competing interests that could be perceived to bias this work—acknowledging all financial support and any other relevant financial or non-financial competing interests.<br><br>This statement is required for submission and will appear in the published article if the submission is accepted. Please make sure it is accurate and that any funding sources listed in your Funding Information later in the submission form are also declared in your Financial Disclosure statement.<br><br>View published research articles from PLOS ONE for specific examples.	The authors have declared that no competing interests exist.

Powered by Editorial Manager® and ProduXion Manager® from Aries Systems Corporation

NO authors have competing interests<br><br>Enter: The authors have declared that no competing interests exist.<br><br>Authors with competing interests<br><br>Enter competing interest details beginning with this statement:<br><br>I have read the journal's policy and the authors of this manuscript have the following competing interests: [insert competing interests here]<br><br>* typeset	
Ethics Statement<br><br>Enter an ethics statement for this submission. This statement is required if the study involved:<br><br>• Human participants<br>• Human specimens or tissue<br>• Vertebrate animals or cephalopods<br>• Vertebrate embryos or tissues<br>• Field research<br><br>Write "N/A" if the submission does not require an ethics statement.<br><br>General guidance is provided below. Consult the submission guidelines for detailed instructions. Make sure that all information entered here is included in the Methods section of the manuscript.	Owing to the systematic review design, ethical approval is not required.

Powered by Editorial Manager® and ProduXion Manager® from Aries Systems Corporation

Format for specific study types<br><br>Human Subject Research (involving human participants and/or tissue)<br>• Give the name of the institutional review board or ethics committee that approved the study<br>• Include the approval number and/or a statement indicating approval of this research<br>• Indicate the form of consent obtained (written/oral) or the reason that consent was not obtained (e.g. the data were analyzed anonymously)<br><br>Animal Research (involving vertebrate animals, embryos or tissues)<br>• Provide the name of the Institutional Animal Care and Use Committee (IACUC) or other relevant ethics board that reviewed the study protocol, and indicate whether they approved this research or granted a formal waiver of ethical approval<br>• Include an approval number if one was obtained<br>• If the study involved non-human primates, add additional details about animal welfare and steps taken to ameliorate suffering<br>• If anesthesia, euthanasia, or any kind of animal sacrifice is part of the study, include briefly which substances and/or methods were applied<br><br>Field Research<br><br>Include the following details if this study involves the collection of plant, animal, or other materials from a natural setting:<br>• Field permit number<br>• Name of the institution or relevant body that granted permission	
Data Availability<br><br>Authors are required to make all data underlying the findings described fully available, without restriction, and from the time of publication. PLOS allows rare exceptions to address legal and ethical concerns. See the PLOS Data Policy and FAQ for detailed information.	Yes - all data are fully available without restriction

Powered by Editorial Manager® and ProduXion Manager® from Aries Systems Corporation

A Data Availability Statement describing where the data can be found is required at submission. Your answers to this question constitute the Data Availability Statement and will be published in the article, if accepted.<br><br>Important: Stating 'data available on request from the author' is not sufficient. If your data are only available upon request, select 'No' for the first question and explain your exceptional situation in the text box.<br><br>Do the authors confirm that all data underlying the findings described in their manuscript are fully available without restriction?	
Describe where the data may be found in full sentences. If you are copying our sample text, replace any instances of XXX with the appropriate details.<br><br>• If the data are held or will be held in a public repository, include URLs, accession numbers or DOIs. If this information will only be available after acceptance, indicate this by ticking the box below. For example: All XXX files are available from the XXX database (accession number(s) XXX, XXX.).<br>• If the data are all contained within the manuscript and/or Supporting Information files, enter the following: All relevant data are within the manuscript and its Supporting Information files.<br>• If neither of these applies but you are able to provide details of access elsewhere, with or without limitations, please do so. For example:<br><br>Data cannot be shared publicly because of [XXX]. Data are available from the XXX Institutional Data Access / Ethics Committee (contact via XXX) for researchers who meet the criteria for access to confidential data.<br><br>The data underlying the results presented in the study are available from (include the name of the third party	All relevant data are within the manuscript and its Supporting Information files.

and contact information or URL).<br>• This text is appropriate if the data are owned by a third party and authors do not have permission to share the data.<br><br>* typeset	
Additional data availability information:	

Powered by Editorial Manager® and ProduXion Manager® from Aries Systems Corporation

Manuscript

Click here to access/download;Manuscript;manuscript_24aug2023.docx

Effect modification of diabetic status on the association between exposure to particulate matter and cardiac arrhythmias: a systematic review and meta-analysis

Kiattichat Tassanaviroj[1], Pimchanok Plodphai[1], Pakpoom Wongyikul[2*¶], Krittai Tanasombatkul[2,3], Krekwit Shinlapawittayatorn[4], Phichayut Phinyo[2,3*¶]

[1] Faculty of Medicine, Chiang Mai University, Thailand

[2] Center for Clinical Epidemiology and Clinical Statistics, Faculty of Medicine, Chiang Mai University, Thailand

[3] Department of Family Medicine, Faculty of Medicine, Chiang Mai University, Thailand

[4] Department of Physiology, Faculty of Medicine, Chiang Mai University, Thailand

[*]Co-corresponding authors

Email: phichayutphinyo@gmail.com (PP), aumkidify@gmail.com (PW)

¶ These authors contributed equally to this work.

Abstract

Particulate matter (PM) leads to numerous health effects including cardiac arrhythmias. An increasing proportion of the population around the world ~~were~~ has been diagnosed with diabetes mellitus (DM). Exposure to PM and DM have been associated with an increased ~~the~~ risk of cardiovascular diseases. Thus, the objective of this study is to investigate whether PM ~~associates~~ is associated with cardiac arrhythmias and whether DM status ~~modify~~ modifies its effect. The search was done on Pubmed and Embase until 18 January 2023. We included cohort and case-crossover studies ~~which~~ that showed the effect of PM on cardiac arrhythmias and the effect modification of DM status. We used the DerSimonian and Laird random-effects model to calculate the pooled estimates with their corresponding 95% confidence intervals (CIs). We found 217 studies ~~which~~ that were subsequently screened. Nine studies matched the inclusion criteria and 5 of them were included in meta-analysis. There were 4,431,452 participants, 2,556 of them had DM. We found that PM, both PM2.5 and PM10, showed a significant effect on cardiac arrhythmias (OR 1.10 (95%CI 1.04-1.16)). However, the effect modification of diabetic status was not identified (OR 1.18 (95%CI 1.01-1.38) for the diabetic group and OR 1.08 (95%CI 1.02-1.14) for the non-diabetic group) (p-value of subgroup difference=0.304). In conclusion, PM is associated with cardiac arrhythmias, but DM status may not modify its effect. The important limitation was the comparability of studies. Further study with good design and methodology is required.

Commented [Ma1]: Insert article 'an'

Commented [Ma2]: Introduction well summarized

Introduction

Particulate matter (PM) contributes to many health effects including cardiac arrhythmias [1]. Many *in vitro* studies showed that PM produced adverse effects on cell survival, inflammation, DNA damage, mitochondrial damage, and oxidative stress [1-8]. Moreover, *in vivo* studies found that PM significantly affects inflammation, DNA damage, and oxidative stress [2, 3, 7-11]. There ~~are~~ is some evidence that PM associated with cardiac arrhythmia both in experimental animals and humans [12-14]. According to previous studies, PM was associated with cardiac arrhythmias in humans, both healthy and unhealthy persons. Additionally, several studies were performed to investigate the effect of PM on cardiac arrhythmias, and some of these continued studying to identify the diseases or conditions that were sensitive to the effect of PM such as hypertension and diabetes mellitus (DM).

There were many diseases associated with PM and cardiac arrhythmias including DM. Specifically, DM increased inflammation and oxidative stress, associated with abnormal cardiac electrophysiology and cardiac autonomic neuropathy, which make individuals with the disease susceptible to cardiac arrhythmias [15, 16]. PM ~~increased~~ increases the risk of cardiac arrhythmia ~~both~~ in DM-induced rats and diabetic patients [12, 17]. According to ~~these~~ this evidence, we hypothesized that individuals with DM are the susceptible group ~~for~~ to PM-associated cardiac arrhythmias [12, 15-17].

~~There were meta-analyses~~ Some meta-analyses investigated the association between PM and cardiac arrhythmias, but almost all of them focused on atrial fibrillation (AF) and all of them did not perform subgroup analysis of diabetic status [14, 18-21]. Only one meta-analysis was conducted to find the association between PM and AF with the subgroup analysis, they found that exposure to PM was associated with the onset of AF episode but the modifying effect of diabetic status was not observed [22]. We then aimed to evaluate the effect of particulate matter on any cardiac arrhythmias and to compare the occurrence of any cardiac arrhythmias in the presence of particulate matter between diabetic and non-diabetic patients.

Method

Eligibility criteria

Studies were included in our systematic review based on the following criteria: (1) a prospective or retrospective longitudinal study (e.g., cohort or case-control); (2) population with and without DM and report the outcome data separately; (3) PM as exposure of interest; and (4) report the occurrence of any cardiac arrhythmias (e.g., atrial fibrillation) as one of the outcomes. The studies were included in the meta-analysis if they provided the odds ratios as the outcome statistic.

Studies were excluded based on the following criteria: (1) published studies in languages other than English; (2) studies without abstract and/or available full text; and (3) duplicated studies.

Information sources and search strategy

A pre-specified search strategy was employed for searching for literature until the end of December 2022. Electronic medical and scientific databases included were PubMed/MEDLINE,

and EMBASE. The key search terms were “Diabetes mellitus”, “Particulate matter”, and “cardiac arrhythmias”. A search for grey literature or non-published studies ~~were~~ was directed through the Clinical Trial Registry and Google ~~scholar~~Scholar. The authors also reviewed the list of references from systematic reviews and/or meta-analyses with related topics. Relevant studies identified from reference reviews but not included from previously mentioned electronic database searches would also be included at this point. An updated search was conducted on 18 January 2023, before the final analyses and formal dissemination, by using the same set of search terms as ascertained in the pre-specified search strategies.

Study selection

We searched the titles and abstracts of relevant literature from the databases until 18 January 2023. Two investigators (K.T. and P.P.) independently screened records for relevant evidence using Rayyan, a web application to screen systematic review records. Any disagreement during the screening procedures ~~were~~ was resolved by consulting with an expert in cardiac electrophysiology (K.S.).

Data extraction

Two authors (K.T. and P.P.) extracted the information as follows: study authorship, year of publication, study period, country/location, study design, patient demographics including mean age, sample size, types of particulate matter, types of effect (i.e., short, or long term), outcomes (i.e., hospital admission, or ER visit), and Odds ratio. Any disagreement during the process was resolved by discussion with the cardiac electrophysiology expert (K.S.). For studies with incomplete information, we contacted the authors of those studies via email. If they did not respond within 2 weeks, the data would be imputed or reported as missing.

Risk of bias assessment

Two authors (K.T. and P.P.) autonomously checked the quality of each study ~~included~~including using the Newcastle-Ottawa Scale (NOS) quality assessment tool. NOS assessed the quality of study and bias based on three separate domains, which are subject selection, comparability, and outcome assessment. ~~In accordance with~~ Following the number of stars received in each domain, studies will be appraised as good, fair, or poor quality. Studies with 3 to 4 stars in the selection domain, 1 to 2 stars in the comparability domain~~,~~ and 2 to 3 stars in the outcome domain will be appraised as good quality. Studies with 2 stars in the selection domain, 1 to 2 stars in the comparability domain, and 2 to 3 stars in the outcome domain will be appraised as fair quality. Studies with 0 to 1 stars in the selection domain, ~~or~~ 0 stars in the comparability domain, or 0 to 1 stars in the outcome domain will be appraised as poor quality. Any disagreements were resolved during the quality assessment process by consulting with the expert in cardiac electrophysiology (K.S.) and the clinical methodologist (P.P.).

Commented [Ma3]: This may be too wordy

Synthesis method

A traditional approach of a pairwise meta-analysis ~~were~~ was used for data synthesis. The statistical heterogeneity will be assessed by the use of the Cochrane Q test and I-squared statistics.

As all studies are expected to carry clinical and methodological heterogeneity, we used the DerSimonian and Laird random-effects model to estimate the pooled odds ratio with their 95% confidence intervals (CIs). Studies without odds ratio were not included in data synthesis. We performed subgroup analysis of diabetic status to investigate whether diabetic status ~~modify~~ modifies the effect of the association between particulate matter exposures and cardiac arrhythmias.

This systematic review and meta-analysis ~~was~~ were guided and reported in concordance with the Cochrane Handbook of Systematic Review and the Preferred Reporting Items for Systematic Reviews and Meta-analyses (PRISMA), respectively. All analyses were conducted using Stata 17 (StataCorp, College Station, Texas, USA). P-values <0.05 ~~was~~ were regarded as statistically significant. This systematic review and meta-analysis was registered in PROSPERO. The record ID is CRD42023407589.

Results

Study selection

The search from databases and citations found 215 records~~,~~ and 44 duplications were removed. The titles and abstracts were screened, and 159 records were excluded. We screened the full~~-~~ text of the 12 remaining records, 1 of them ~~were~~ was excluded due to study design which not suitable with our inclusion criteria and 2 of them did not provide usable data. ~~9~~ Nine remaining studies were included in our systematic review (23-31), and 5 of them were included in our meta-analysis [23, 24, 27, 29, 30]. (Fig 1)

Commented [Ma4]: A number less than ten is better stated in words

Fig 1: PRISMA Flow diagram

Study characteristics

The systematic review included a total of 9 studies. Two studies were conducted in Taiwan, two were conducted in the USA, two were conducted in China, one was conducted in Georgia, one was conducted in South Korea, and one was conducted in Sweden. Five studies were case-crossover studies~~,~~ and 4 studies were cohort studies. Four studies focused on the effect of PM2.5, two studies focused on the effect of PM10, and three studies focused both on PM2.5 and PM10. Seven studies focused on the short-term effect of PM, and two studies focused on the long-term effect. The summary of the studies ~~are~~ is presented in Table 1.

Table 1: Summary of included studies

Author	Period	Country/location	Study design	PM	Outcome	Short/long term	N	N (DM)	Mean age	Outcome for DM group	Outcome for non DM group

									(ye ar)		
Peel (2006)	1993-2000	Georgia	Case-crossover	10	ER visits	Short term	4,407,535	1,562	N/A	OR=1.049 (0.968, 1.137)	OR=1.009 (0.989, 1.029)
Chiu (2009)	2000-2006	Taiwan	Case-crossover	10	Hospital admission	Short term	21,581	452	N/A	OR=0.95 (0.56, 1.63)	OR=1.08 (1.04, 1.13)
Zanobetti (2014)	2000-2010	USA	Cohort	2.5	EKG measurement	Short term	1,448	305	75.3	OR=1.54 (1.06, 2.4)	OR=1.34 (1.16, 1.64)
Zheng (2018)	2014-2015	China	Case-crossover	2.5	Hospital admission	Short term	175,265	19,947	N/A	Percent change= 4.93 (2.51, 7.34)	Percent change= 1.88 (1.07, 2.78)
				10						Percent change= 7.16 (4.03, 10.30)	Percent change= 2.69 (1.52, 3.85)
Kim (2019)	2009-2013	South Korea	Cohort	2.5	Outpatient hospital visits or hospital admissions	Long term	432,587	27,627	N/A	HR=1.192 (1.179, 1.205)	HR=1.181 (1.177, 1.185)
				10						N/A	N/A
Lee (2019)	2006-2011	Taiwan	Case-crossover	2.5	Hospital admission	Short term	670	213	70.5	OR=1.34 (1.02, 1.86)	OR=1.2 (0.81, 1.28)
Dahlquist (2020)	2012-2013 and 2016-2018	Sweden	Cohort	2.5	EKG measurement	Short term	218	24	N/A	OR=0.98 (0.78, 1.24)	OR=1.06 (0.98, 1.15)
				10						OR=1.48 (1.14, 1.92)	OR=1.08 (0.99, 1.18)
Liang (2021)	2011-2014	China	Case-crossover	2.5	ER visits	Short term	8,241	N/A	60	Relative risk=1.25	Relative risk=14

Jin (2022)	2000-2016	USA	Cohort	2.5	All available Medicare claims	Long term	32,165,933	N/A	N/A	HR=1.010 (1.008, 1.011)	HR=1.003 (1.002, 1.004)

There were 7 studies focused on the effect of PM2.5 on cardiac arrhythmias which had subgroup analysis of diabetic status [24-28, 30, 31]. Six studies showed that PM2.5, DM, and cardiac arrhythmias were associated. Only the study by Dahlquist and colleagues showed that these factors were not associated.

There were 5 studies focused on the effect of PM10 on cardiac arrhythmias which subgroup analysis of diabetic status was done [23, 24, 26, 29, 31]. Three studies showed that PM10, DM, and cardiac arrhythmias were associated. Only the study by Chiu and colleagues showed that these factors were not associated.

According to 5 studies ~~which~~ that were included in our meta-analysis, there were 4,431,452 participants included in the meta-analysis, ~~which~~ 2,556 of ~~them~~ whom are DM patients. The average age of participants was 74 years old. There were different outcome measurements in each ~~studies~~ study: two were hospital admissions for cardiac arrhythmias, two were ECG measurements, and one was ER visits for cardiac arrhythmias.

Risk of bias in studies

Of those 9 studies included in our meta-analysis, four were good quality, three were fair quality, and two were poor quality. The comparability of the studies tends to have bias which might affect the meta-analysis outcome. The risk of bias assessments ~~was~~ is presented in Table 2.

Table 2: Risk of bias

Author (year)	Selection				Comparability	Outcome			Total score	Quality of study
	Representativeness of the exposed cohort	Selection of the non-exposed cohort	Ascertainment of exposure	Demonstration that outcome of interest was not present at start of study		Assessment of outcome	Was follow-up long enough for outcomes to occur	Adequacy offollow up of cohorts		
Peel et al. (2006)	*	*	*		*	*	*		6	Good
Chiu et al. (2009)	*	*	*	*	*	*	*		7	Good
Zanobetti et al. (2014)			*	*		*	*	*	5	Poor
Zheng et al. (2018)	*	*	*		*	*	*		6	Good
Kim et al. (2019)	*	*	*	*		*	*		6	Fair
Lee et al. (2019)	*	*	*	*	*	*	*		7	Good
Dahlquist et al. (2020)	*	*	*	*	*			*	6	Fair
Liang et al. (2021)	*	*	*		*	*			5	Poor
Jin et al. (2022)	*	*	*	*		*	*		6	Fair

The association between PM and cardiac arrhythmias

Both PM2.5 and PM10, were associated with the occurrence of cardiac arrhythmias in the study population (OR 1.10 (95%CI 1.01, 1.38), p<0.001). The meta-analysis showed that DM status does not modify the effect (OR of DM group 1.08 (95%CI 1.02, 1.14), p<0.001, OR of non-DM group 1.18 (95%CI 1.01, 1.38), p=0.041). This showed that there was no significant difference between the two groups (p=0.304) but the DM group tended to have a larger effect size than the non-DM group. (Fig 2)

Fig 2: The association between PM and cardiac arrhythmias

The association between PM2.5 and cardiac arrhythmias

Three studies with 2,336 participants focused on PM2.5. It showed a significant effect on cardiac arrhythmias in the study population (OR 1.18 (95%CI 1.04, 1.34), p=0.008). PM2.5

showed a significant effect on cardiac arrhythmias in the non-DM group (OR 1.18 (95%CI 1.00, 1.38), p=0.045), but showed a non-significant effect on cardiac arrhythmias in the DM group (OR 1.22 (95%CI 0.93, 1.60), p=0.092). DM status did not modify the effect (I^2 = 55.63%, test of group differences: Q_b (1) = 0.06, p=0.813), but showed the trend of larger effect size than the non-DM group. (Fig 3)

Fig 3: The association between PM2.5 and cardiac arrhythmias

The association between PM10 and cardiac arrhythmias

Three studies with 4,429,334 participants focused on PM10. It showed a significant effect on cardiac arrhythmias in the study population (OR 1.06 (95%CI 1.01, 1.12), p=0.028). PM10 showed a non-significant effect on cardiac arrhythmias in both non-DM groups (OR 1.05 (95%CI 0.99, 1.11), p=0.090), and in the DM group (OR 1.16 (95%CI 0.89, 1.51), p=0.261). DM status did not modify the effect between PM10 and the occurrence of cardiac arrhythmias (I^2 = 72.01%, test of group differences: Q_b (1) = 0.56, p = 0.455), but showed the trend of larger effect size than non-DM group. (Fig 4)

Fig 4: The association between PM10 and cardiac arrhythmias

The effect of study quality on the association between PM and cardiac arrhythmias

The quality of each study affected the association between PM and cardiac arrhythmias. Poor quality ~~study~~ studies (OR 1.37 (95%CI 1.17, 1.61), p<0.001), and fair quality studies (OR 1.10(95%CI 0.99, 1.21), p=0.067) tended to overestimate the association. Good quality studies showed a significant effect of PM on cardiac arrhythmias (OR 1.06 (95%CI 1.00, 1.11), p=0.043). The test for subgroup difference according to study quality showed statistical significance (p=0.010) (Fig 5).

Fig 5: The effect of study quality on the association between PM and cardiac arrhythmias

Discussion

Our results suggested that both PM2.5 and PM10 were associated with cardiac arrhythmias. According to subgroup analysis, diabetic status did not show the effect modification~~,~~ but showed ~~the~~ a higher trend of the effect.

~~There were many previous meta-analyses that~~ Many previous meta-analyses studied about the association between PM and cardiac arrhythmias. They found that PMs were associated with cardiac arrhythmias. Most of them focused on AF, and they did not perform~~ed~~ a subgroup analysis of diabetic status [14, 18-21].

Commented [Ma5]: This sentence may seem too wordy

The underlying mechanisms of the association between PM and cardiac arrhythmias in diabetic patients are still ambiguous. A study by Schneider and colleagues found that PM2.5 caused systemic inflammation and altered ventricular repolarization in diabetic individuals which might lead to cardiac arrhythmias [17]. ~~Basically, t~~ There are elevated inflammation and oxidative

stress, abnormal cardiac electrophysiology, and cardiac autonomic neuropathy in diabetic patients, which make them already susceptible to cardiac arrhythmias [15, 16]. By adding the effect of PM on these factors, we can infer that PM and diabetic status are related ~~in order~~ to cause cardiac arrhythmias. In contrast to this hypothesis, our results showed that diabetic status did not modify the effect of PM on cardiac arrhythmias.

Our results indicated that PM, both PM2.5 (OR = 1.18 (1.04, 1.34)) and PM10 (OR = 1.06 (1.01, 1.12)), were associated with the occurrence of cardiac arrhythmias. This is agreeable with the prior studies. The study by Yue and colleagues showed that PM2.5 and PM10 were associated with atrial fibrillation episodes, whether short or long-term ~~of~~ exposure. Although the results were in the same direction, it focused only on atrial fibrillation, which is one of cardiac arrhythmias [22]. The study by Song and colleagues showed that both PM2.5 and PM10 were associated with hospitalization and mortality due to cardiac arrhythmias which is consistent with our results [14].

Our results showed that diabetic status did not modify the effect of PM on cardiac arrhythmias, which is contrary to our hypothesis. The results were in the same direction ~~with~~ as the prior systematic review and meta-analysis. The study by Yue and colleagues indicated in the subgroup analysis that diabetic status did not show an~~d~~ effect on the association between PM and atrial fibrillation [22]. Although the results were consistent, they were opposed ~~with~~ to the prior study by Schneider and colleagues that PM led to cardiac arrhythmias in diabetic individuals [17]. This may be due to the comparability.

Cardiac arrhythmias are multifactorial, which means there can be other factors than DM that can contribute to the symptom occurrence and show confounding effects. Our main limitation is the comparability of the included evidence~~s~~. First, some studies indicated that their results were associated with PM, but there ~~were~~ was no evidence~~s~~ that the effect from other pollutants ~~were~~ was adjusted. This may lead to overestimation in some studies. Second, most participants in all ~~study~~ studies had comorbidities or some condition such as hypertension, or congestive heart failure. When subgroup analysis of diabetic status was done, the non-DM cohort might ~~be~~ have been patients with comorbidities, rather than healthy subjects, which might ~~showed~~ have shown the confounding effect, making the effect of diabetic status ~~being~~ underestimated. Further study with a low risk of bias in the comparability domain is required.

Medication used by ~~the~~ DM patients could be another possible reason for the irrelevant result. There were some studies indicated that some antidiabetic drugs showed ~~the~~ a protective effect of cardiac arrhythmias. The network meta-analysis conducted by Shi and colleagues showed that anti-diabetic drugs could lower the risk of AF in DM patients, and glucagon-like peptide-1 receptor agonist was the most effective among the medications [32]. The nationwide cohort study in South Korea conducted by Kim and colleagues showed that metformin and thiazolidinedione could prevent AF in DM patients [33].

There were some limitations in the reviewing process. We reviewed only studies ~~which~~ that showed the effect of PM on cardiac arrhythmias with subgroup analysis of DM status. The studies ~~which~~ that did not perform subgroup analysis of DM were not included, so the results in part of the effect of PM

on cardiac arrhythmias were synthesized from smaller sample sizes than in reality. Future meta-analyses should ~~conduct the~~ search for the studies with and without subgroup analysis of DM.

The studies in the future should directly examine the effect modification from specific subgroups (e.g. DM). They should properly adjust the confounding effect~~,~~ since cardiac arrhythmias are multifactorial. Collecting information ~~of~~ on all comorbidities each participant~~s~~ have, the medication they use, and their family history ~~about~~ of cardiac arrhythmias might be some examples to handle ~~with~~ the confounding factors.

Conclusion

Our systematic review and meta-analysis found that PM, both PM2.5 and PM10, had a significant effect on cardiac arrhythmia. However, diabetic status did not aggravate its deleterious effects~~,~~ but showed ~~the~~ a higher trend. Study with high quality, especially ~~on~~ in the comparability domain, is required, and political policies should focus more on the harmful effects of PMs.

Financial support

There ~~were~~ was no financial support for this meta-analysis.

Competing interests

There were no competing interests.

References

1. Cao J, Qin G, Shi R, Bai F, Yang G, Zhang M, et al. Overproduction of reactive oxygen species and activation of MAPKs are involved in apoptosis induced by PM2.5 in rat cardiac H9c2 cells. J Appl Toxicol. 2016;36(4):609-17.
2. Feng L, Wei J, Liang S, Sun Z, Duan J. miR-205/IRAK2 signaling pathway is associated with urban airborne PM(2.5)-induced myocardial toxicity. Nanotoxicology. 2020;14(9):1198-212.
3. Jiang J, Liang S, Zhang J, Du Z, Xu Q, Duan J, et al. Melatonin ameliorates PM(2.5) -induced cardiac perivascular fibrosis through regulating mitochondrial redox homeostasis. J Pineal Res. 2021;70(1):e12686.
4. Luo CM, Feng J, Zhang J, Gao C, Cao JY, Zhou GL, et al. 1,25-Vitamin D3 protects against cooking oil fumes-derived PM2.5-induced cell damage through its anti-inflammatory effects in cardiomyocytes. Ecotoxicol Environ Saf. 2019;179:249-56.
5. Qi Z, Song Y, Ding Q, Liao X, Li R, Liu G, et al. Water soluble and insoluble components of PM(2.5) and their functional cardiotoxicities on neonatal rat cardiomyocytes in vitro. Ecotoxicol Environ Saf. 2019;168:378-87.
6. Yang X, Feng L, Zhang Y, Hu H, Shi Y, Liang S, et al. Cytotoxicity induced by fine particulate matter (PM(2.5)) via mitochondria-mediated apoptosis pathway in human cardiomyocytes. Ecotoxicol Environ Saf. 2018;161:198-207.
7. Yang X, Zhao T, Feng L, Shi Y, Jiang J, Liang S, et al. PM(2.5)-induced ADRB2 hypermethylation contributed to cardiac dysfunction through cardiomyocytes apoptosis via PI3K/Akt pathway. Environ Int. 2019;127:601-14.
8. Zou L, Li B, Xiong L, Wang Y, Xie W, Huang X, et al. Urban fine particulate matter causes cardiac hypertrophy through calcium-mediated mitochondrial bioenergetics dysfunction in mice hearts and human cardiomyocytes. Environ Pollut. 2022;305:119236.

9. Hu X, Chen M, Cao X, Yuan X, Zhang F, Ding W. TGF-β-Containing Small Extracellular Vesicles From PM(2.5)-Activated Macrophages Induces Cardiotoxicity. Front Cardiovasc Med. 2022;9:917719.

10. Yin B, Zhang X, Ren J, Chen F, Liang J, Zhang H, et al. The protective effects of procyanidin supplementation on PM(2.5)-induced acute cardiac injury in rats. Environ Sci Pollut Res Int. 2023;30(4):10890-900.

11. Zhao L, Zhang L, Chen M, Dong C, Li R, Cai Z. Effects of Ambient Atmospheric PM(2.5), 1-Nitropyrene and 9-Nitroanthracene on DNA Damage and Oxidative Stress in Hearts of Rats. Cardiovasc Toxicol. 2019;19(2):178-90.

12. Carll AP, Crespo SM, Filho MS, Zati DH, Coull BA, Diaz EA, et al. Inhaled ambient-level traffic-derived particulates decrease cardiac vagal influence and baroreflexes and increase arrhythmia in a rat model of metabolic syndrome. Part Fibre Toxicol. 2017;14(1):16.

13. Nadziejko C, Fang K, Narciso S, Zhong M, Su WC, Gordon T, et al. Effect of particulate and gaseous pollutants on spontaneous arrhythmias in aged rats. Inhal Toxicol. 2004;16(6-7):373-80.

14. Song X, Liu Y, Hu Y, Zhao X, Tian J, Ding G, et al. Short-Term Exposure to Air Pollution and Cardiac Arrhythmia: A Meta-Analysis and Systematic Review. Int J Environ Res Public Health. 2016;13(7).

15. Abuelgasim E, Shah S, Abuelgasim B, Soni N, Thomas A, Elgasim M, et al. Clinical overview of diabetes mellitus as a risk factor for cardiovascular death. Rev Cardiovasc Med. 2021;22(2):301-14.

16. Karam BS, Chavez-Moreno A, Koh W, Akar JG, Akar FG. Oxidative stress and inflammation as central mediators of atrial fibrillation in obesity and diabetes. Cardiovasc Diabetol. 2017;16(1):120.

17. Schneider A, Neas LM, Graff DW, Herbst MC, Cascio WE, Schmitt MT, et al. Association of cardiac and vascular changes with ambient PM2.5 in diabetic individuals. Part Fibre Toxicol. 2010;7:14.

18. Chen M, Zhao J, Zhuo C, Zheng L. The Association Between Ambient Air Pollution and Atrial Fibrillation. Int Heart J. 2021;62(2):290-7.

19. Shao Q, Liu T, Korantzopoulos P, Zhang Z, Zhao J, Li G. Association between air pollution and development of atrial fibrillation: A meta-analysis of observational studies. Heart Lung. 2016;45(6):557-62.

20. Wang F, Ahat X, Liang Q, Ma Y, Sun M, Lin L, et al. The relationship between exposure to PM(2.5) and atrial fibrillation in older adults: A systematic review and meta-analysis. Sci Total Environ. 2021;784:147106.

21. Yue C, Yang F, Wang L, Li F, Chen Y. Association between fine particulate matter and atrial fibrillation in implantable cardioverter defibrillator patients: a systematic review and meta-analysis. J Interv Card Electrophysiol. 2020;59(3):595-601.

22. Yue C, Yang F, Li F, Chen Y. Association between air pollutants and atrial fibrillation in general population: A systematic review and meta-analysis. Ecotoxicol Environ Saf. 2021;208:111508.

23. Chiu HF, Yang CY. Air pollution and emergency room visits for arrhythmias: are there potentially sensitive groups? Journal of toxicology and environmental health Part A. 2009;72(13):817-23.

24. Dahlquist M, Frykman V, Kemp-Gudmunsdottir K, Svennberg E, Wellenius GA, L S Ljungman P. Short-term associations between ambient air pollution and acute atrial fibrillation episodes. Environment international. 2020;141:105765.

25. Jin T, Di Q, Réquia WJ, Danesh Yazdi M, Castro E, Ma T, et al. Associations between long-term air pollution exposure and the incidence of cardiovascular diseases among American older adults. Environment International. 2022;170.

26. Kim IS, Yang PS, Lee J, Yu HT, Kim TH, Uhm JS, et al. Long-term exposure of fine particulate matter air pollution and incident atrial fibrillation in the general population: A nationwide cohort study. Int J Cardiol. 2019;283:178-83.

27. Lee HH, Pan SC, Chen BY, Lo SH, Guo YL. Atrial fibrillation hospitalization is associated with exposure to fine particulate air pollutants. Environmental Health: A Global Access Science Source. 2019;18(1).

28. Liang B, He X, Du X, Liu X, Ma C. Effect of Air Quality on the Risk of Emergency Room Visits in Patients With Atrial Fibrillation. Front Cardiovasc Med. 2021;8:672745.
29. Peel JL, Metzger KB, Klein M, Flanders WD, Mulholland JA, Tolbert PE. Ambient air pollution and cardiovascular emergency department visits in potentially sensitive groups. American Journal of Epidemiology. 2007;165(6):625-33.
30. Zanobetti A, Coull BA, Gryparis A, Kloog I, KloogSparrow D, Vokonas PS, et al. Associations between arrhythmia episodes and temporally and spatially resolved black carbon and particulate matter in elderly patients. Occupational and Environmental Medicine. 2014;71(3):201-7.
31. Zheng Q, Liu H, Zhang J, Chen D. The effect of ambient particle matters on hospital admissions for cardiac arrhythmia: A multi-city case-crossover study in China. Environmental Health: A Global Access Science Source. 2018;17(1).
32. Shi W, Zhang W, Zhang D, Ren G, Wang P, Gao L, et al. Comparison of the effect of glucose-lowering agents on the risk of atrial fibrillation: A network meta-analysis. Heart Rhythm. 2021;18(7):1090-6.
33. Kim S, Park SY, Kim B, Min C, Cho W, Yon DK, et al. Association between antidiabetic drugs and the incidence of atrial fibrillation in patients with type 2 diabetes: A nationwide cohort study in South Korea. Diabetes Res Clin Pract. 2023;198:110626.

Supporting Information

S1 File, PROSPERO protocol registration

Figure 1

Click here to access/download;Figure;Fig1.JPG

Commented [Ma6]: Each figure has to be labelled at the bottom with a title

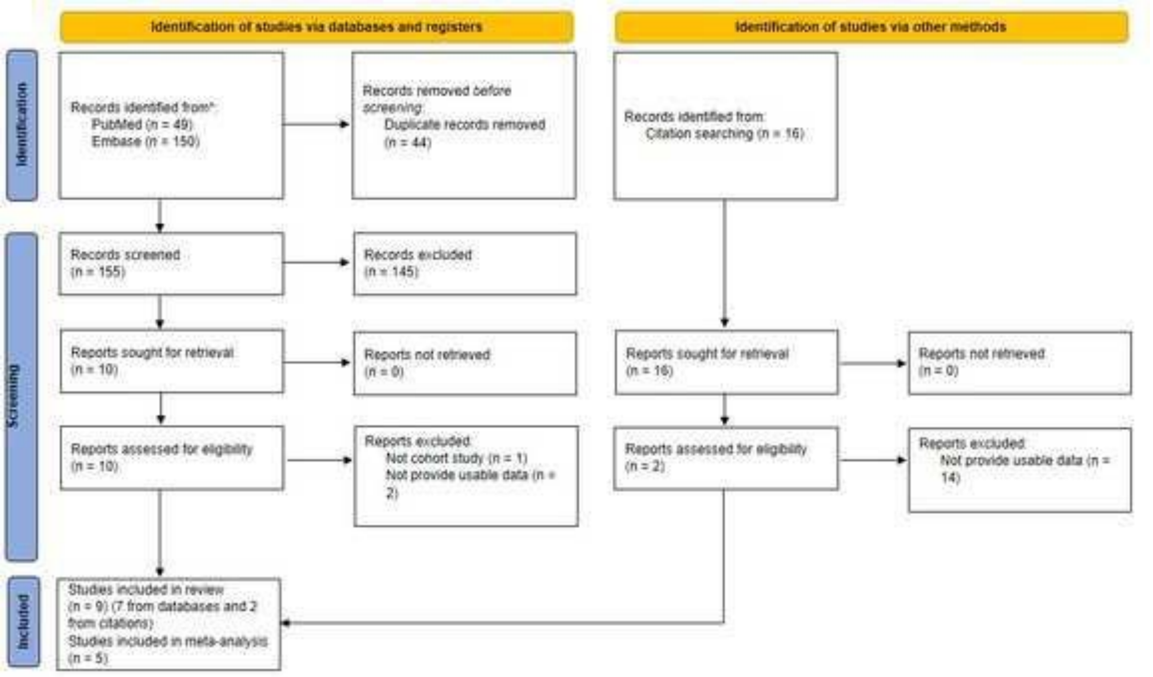

Figure 2

Click here to access/download;Figure;Fig2.png

Commented [Ma7]: Each figure has to be labelled at the bottom with a title

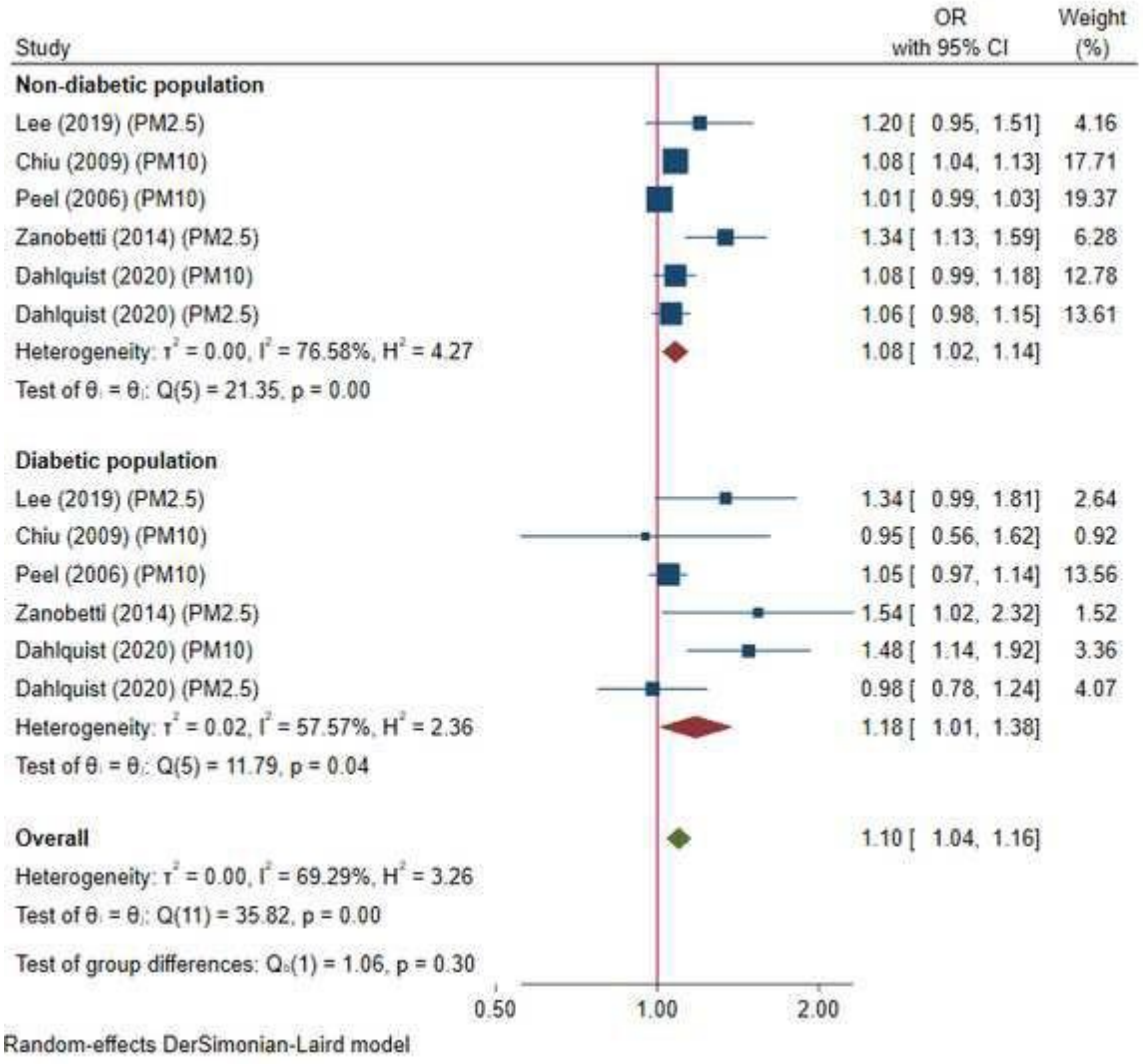

Figure 3

Click here to access/download;Figure;Fig3.png

Commented [Ma8]: Each figure has to be labelled at the bottom with a title

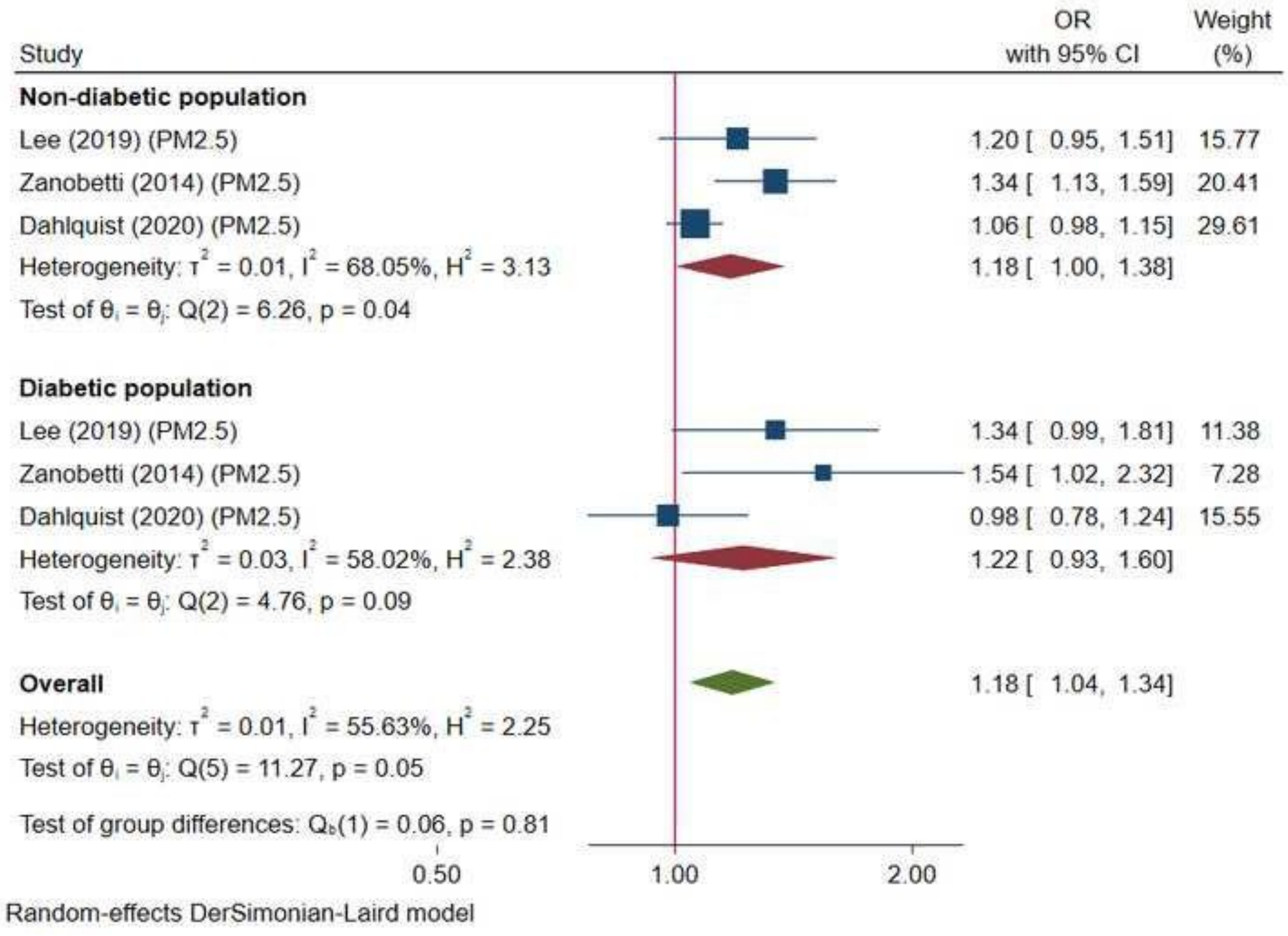

Figure 4

Click here to access/download;Figure;Fig4.png

Commented [Ma9]: Each figure has to be labelled at the bottom with a title

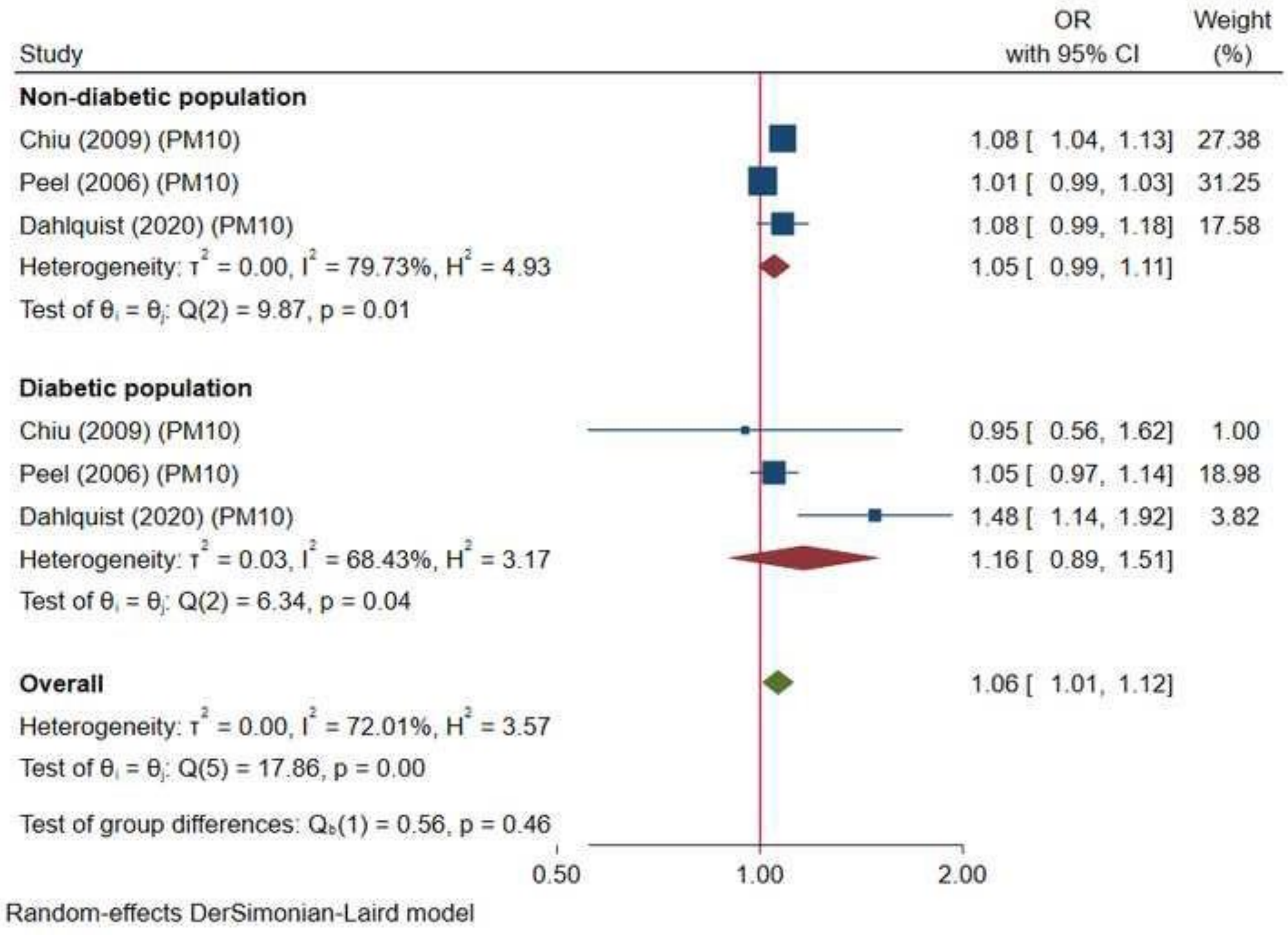

Figure 5

Click here to access/download;Figure;Fig5.png

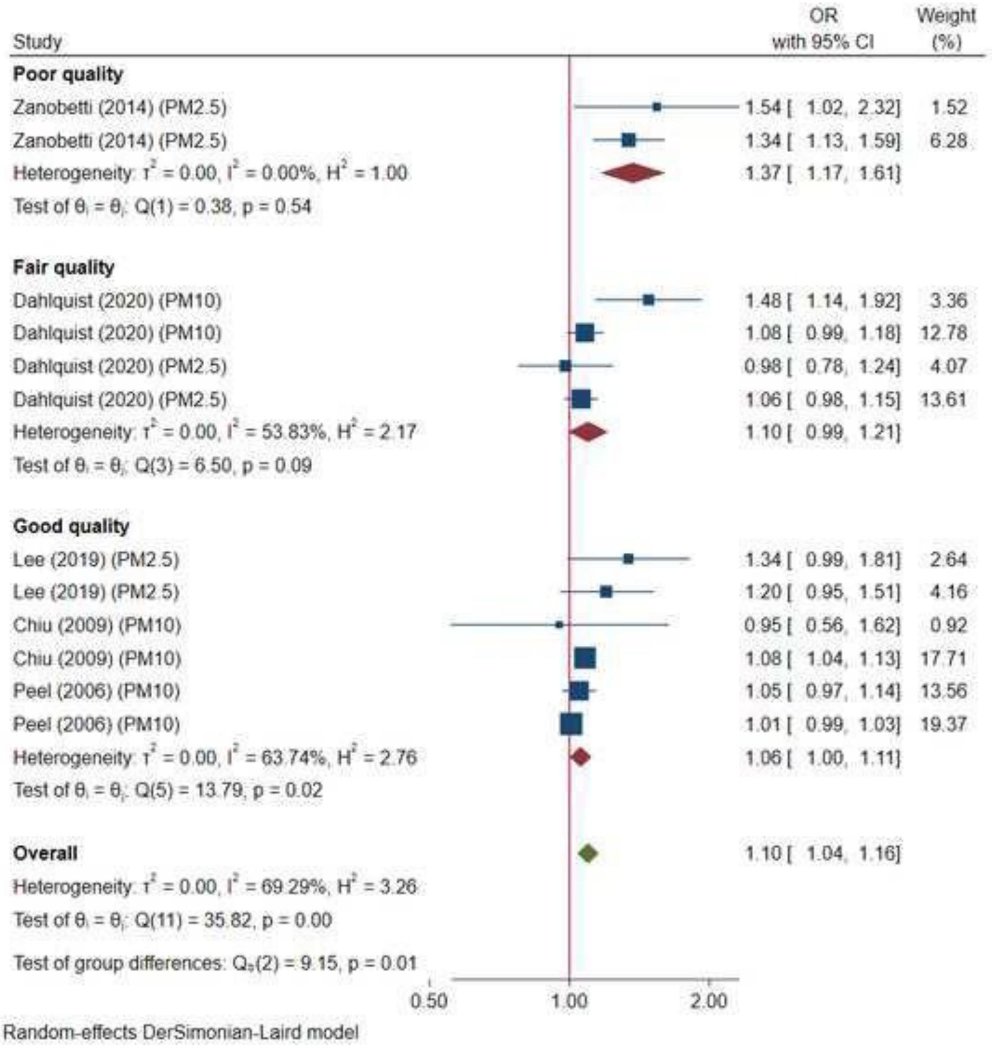

Supporting information

Click here to access/download
Supporting Information
S1_File.pdf
