# Supplementary material for: Effect modification of diabetic status on the association between exposure to particulate matter and cardiac arrhythmias in a general population: A systematic review and meta-analysis
Source: PLoS One. 2024 May 17;19(5):e0301766. doi: 10.1371/journal.pone.0301766 (PMC11101100; doi:10.1371/journal.pone.0301766)
Supplement: Attachment — Submitted filename: Peer Review Comments.docx [file pone.0301766.s004.docx]

## Overall comments

- Overall, the methodological approach and execution of the systematic review and meta-analysis appear sound.
- Good use of Cochrane guidelines and pre-registration of the study.
- The manuscript would benefit from English language copyediting to correct some issues with grammar and flow.
- The small number of studies for meta-analysis (3 each for PM2.5 and PM10) calls into question the use of random effects model and whether it is sufficiently powered; recommend strengthening rationale for use of this model and explanation of the power expectations. (Although I^2^ is appropriately used to assess heterogeneity, please note the Cochrane caution, “Authors should recognize that there is much uncertainty in measures such as I2 and Tau2 when there are few studies. Thus, use of simple thresholds to diagnose heterogeneity should be avoided.”) [[Chapter 10: Analysing data and undertaking meta-analyses | Cochrane Training](https://training.cochrane.org/handbook/current/chapter-10)]. Consider whether the number of studies is sufficient to justify the meta-analysis or if it should be dropped (for example, Chiu’s 1% weight in PM10 analysis is a vanishingly small contribution).
- The reporting of results is generally good, but when reporting the effects of *both* PM10 and PM2.5 on arrythmias, sometimes the language is unclear. Please specify how particulate exposure was combined when reporting these together. Furthermore, the manuscript would be strengthened by explaining why combining PM10 and PM2.5 results makes sense in the first place.
- Publication bias is a notorious potential issue in meta-analysis; although the authors discuss searching for “grey literature,” it is not clear whether such studies were uncovered and how they were handled to eliminate bias. A funnel plot would be useful to elucidate.
- The manuscript would benefit from a discussion of type 1 & 2 DM, whether they play differing roles in PM effects, and how this is reflected (or deemed irrelevant) in these findings.
- Methodologically, the use of imputation and final missingness are an important details that receives no detailed description.

## Specific sections

**Abstract and Introduction**

- Line 30: please specify the timeframe in which DM diagnoses have increased
- Line 54: please provide citations for the studies mentioned in “Additionally, several studies were performed to investigate the effect of PM on cardiac arrhythmias, and some of these continued studying to identify the diseases or conditions that were sensitive to the effect of PM such as hypertension and diabetes mellitus (DM).”
- Line 58 Seems like a repeat.
- Line 63: It seems that your hypothesis is that DM diagnosis indicates a *more* susceptible group.
- Line 65: Are you suggesting that AF is not a type of arrhythmia? It is unclear why you highlight this fact.

**Method**

- Line 76: Given different disease trajectory and effects, discuss type 1 versus type 2 diabetes and why you treat them as equivalent.
- Line 85: What became of your grey literature search? Were studies uncovered and included in this analysis? How does your later discussion of bias relate to this search?
- Line 101: Here and elsewhere the “types of effect” should be “type (or length) of exposure”. For reference, see your bibliography for Kim et al (2019) where “long-term” is clearly referring to PM exposure: Kim IS, Yang PS, Lee J, Yu HT, Kim TH, Uhm JS, et al. **Long-term** **exposure** of fine particulate matter air pollution and incident atrial fibrillation in the general population: A nationwide cohort study.
- Line 105: Provide substantially more detail on the methods for imputation, which data were imputed, and what the remaining missingness was. This is an extremely important detail lacking clarity.
- Line 106 and following – risk of bias: Consider the use of a funnel plot to further elucidate bias handling.
- Lines 122,123: Please provide more details on your rationale for using a random effects model. Given the small number of studies used for meta-analysis, it is not clear how you evaluated I^2^ and Q or compared results to a fixed-effects methodology, and whether the small sample size impacts potential odds ratio estimates.

**Results**

- Line 149: Please explain why mixing long-term and short-term exposure durations (what you call “effect”) is appropriate for the meta-analysis.
- Line 169 and Table 1: You mention “9 studies included in the meta-analysis”, but in Figure 1 I see only 6 studies that contribute to findings. (For example, where is Jin et al. in Figure 1?)
- Line 175: please clarify that this section is addressing the effect of both (or either) PM2.5 and PM10 together, whereas the subsequent sections are specific to each pollutant alone.

**Discussion**

- Lines 234,235: Please restate your findings with respect to the effect modification, as you did in the preceding paragraph.
- Line 240: Please expand on the comparability thesis as the reason for differing Schneider results.
- Lines 258-260: The selection criteria are an extremely important point and should probably be included in your abstract, results, and other text that states a finding: “…among PM and cardiac arrythmia studies that include diabetes as a potential modifier, our findings were…”
